# Evaluation and Implication of Case Volume Variation in Level 1 and 2 Trauma Centers

**DOI:** 10.1097/AS9.0000000000000589

**Published:** 2025-06-12

**Authors:** Patrick L. Johnson, Bryant W. Oliphant, Jonathan E. Williams, Cody L. Mullens, Raymond A. Jean, Anne H. Cain-Nielsen, John W. Scott, Mark R. Hemmila

**Affiliations:** From the * Department of Surgery, University of Michigan Medical School, Ann Arbor, MI; † Center for Healthcare Outcomes and Policy, University of Michigan, Ann Arbor, MI; ‡ Department of Orthopaedic Surgery, University of Michigan, Ann Arbor, MI; § Department of Surgery, University of Washington, Harborview Medical Center, Seattle, WA.

**Keywords:** trauma networks, trauma systems

## Abstract

**Objective:**

To evaluate variation in case volume and procedural volume across level 1 and 2 U.S. trauma centers.

**Background:**

When trauma center distribution does not fit regional needs, the longstanding volume-outcomes relationship in trauma care is at risk. Case volume variability has important implications for trauma center distribution, patient outcomes, and clinical skills maintenance.

**Methods:**

We placed trauma centers into quintiles based on average annual patient volume meeting American College of Surgery Trauma Quality Improvement Program (ACS TQIP) inclusion criteria from 2017 to 2021. Patient characteristics and procedures performed were evaluated across case volume and trauma center verification levels. We evaluated the relationship between procedural volume and case volume by examining the number of interventions performed as a proportion of patients with a potential indication.

**Results:**

We identified 1,902,005 patients among 228 level 1 and 288 level 2 trauma centers. A fourfold difference in ACS TQIP qualifying patient volume was present between the highest and lowest quintile level 1 and 2 trauma centers (1888 ± 481 vs 484 ± 109, 966 ± 223 vs 224 ± 70). The lowest quintile centers performed very low volumes of essential trauma procedures including hemorrhage control (22 per year) and pelvic fracture operations (10 per year). Low-volume trauma centers performed proportionally fewer procedures, including hemorrhage control procedures for patients presenting with tachycardia and hypotension (25.9 vs 31.8%, *P* < 0.001).

**Conclusions:**

Trauma center case volume varies widely, with 1-in-5 level 1 trauma centers averaging <2 hemorrhage control procedures per month. Furthermore, low-volume centers perform proportionally fewer procedures suggesting unexplained variation in practice patterns.

## INTRODUCTION

The development of trauma systems, implementation of verification and designation programs, and proliferation of trauma centers have been essential for improving patient access to quality trauma care.^1–5^ However, the lack of a national decision-making body governing trauma center distribution has resulted in regionalized networks where trauma center resources may be mismatched to population needs.^6,7^ Trauma center over-proliferation in areas already adequately served by an existing trauma center can result in a decrease in patient volume and impact the patient volume-outcome relationship.^8^ Lower threshold case volumes and increasing variation between low and high-volume trauma centers have implications for optimal patient outcomes, clinical skills maintenance, and surgical education. There is also an economic tradeoff between local access to broad-spectrum trauma care and efficient deployment of resource-intensive clinical enterprises.

Though specific overall case requirements exist for trauma center verification, the current understanding of variation in case volume and procedural volume among level 1 and 2 trauma centers is incomplete. The American College of Surgeons Committee on Trauma (ACS COT) specifies that an adult trauma center must care for at least 1200 trauma patients meeting National Trauma Data Standard inclusion criteria per year or 240 trauma patients with an injury severity score (ISS) >15 per year to achieve level 1 trauma verification status.^9^ While there are no minimum case volumes required for level 2 trauma centers, both level 1 and 2 trauma centers must fulfill similar staffing, resource, and performance improvement criteria for verification, by the ACS COT Verification, Review, and Consultation Program, and designation, by regional governmental institutions. Despite similar standards for resources and operations across facilities, prior literature has demonstrated that higher-volume trauma centers achieve superior patient outcomes in a variety of domains, including in-hospital mortality.^10–12^ In this context, an improved understanding of case volume variation across U.S. trauma centers has important implications for optimizing trauma systems nationwide.

Leveraging patient and facility data in the ACS Trauma Quality Improvement Program (ACS TQIP) participant use file, this study sought to provide a detailed evaluation of case volume variation among level 1 and 2 trauma centers across the United States. This granular dataset also permits the assessment of various patient-level characteristics, injury patterns, and patient-care interventions. The underlying hypothesis was that case volume and procedural volume will vary widely among level 1 and level 2 trauma centers. Furthermore, we hypothesized that procedural volume will remain proportional to case volume across high and low-volume trauma centers, indicating consistent patterns of care.

## MATERIALS AND METHODS

Data was extracted from the ACS TQIP participant use files for admission years 2017–2021 (Table S1, see https://links.lww.com/AOSO/A513). Inclusion criteria for the dataset are: age ≥16 years, blunt or penetrating mechanism of injury, abuse of any trauma type, at least 1 valid abbreviated injury scale (AIS) 05/08 injury code with severity between 3 and 6, admitted patients, and patients who died in the emergency department (ED).^13^ Patients were excluded if they had a preexisting advanced directive to withhold life-sustaining interventions, met criteria for no signs of life, or all recorded injuries were consistent with severe burns and no other trauma.^14^ Beyond these ACS TQIP inclusion and exclusion criteria, trauma centers were excluded if any of the following occurred: no verification level of 1 or 2 within the time frame being investigated, did not participate in ACS TQIP, or mean annual volume of ≤50 patients age 16 or greater with AIS ≥3 in at least 1 body region.

The mean annual center volume was calculated by dividing the total number of patients by the number of years of data that the center contributed to the dataset. Trauma centers with at least 1 year of data available from 2017 to 2021 were included. Trauma centers were placed into quintiles of overall mean annual volume and were further stratified by trauma center verification level. For trauma centers that changed the verification level within the 2017–2021 time frame, the higher verification level was used.

Using ACS TQIP patient data, ICD-10 procedure and diagnosis codes, and AIS05 codes, flags were created to identify various patient characteristics. Patients were counted with a given characteristic in each year, for each trauma center. The mean, standard deviation, median, and interquartile range of trauma centers’ yearly volumes of patient characteristics within each quintile were then calculated. Comparisons of patient characteristics were performed across quintiles using *χ*^2^ tests or analysis of variance. We also compared patient characteristics between just the lowest quintile (Q1) and the highest quintile (Q5).

To evaluate how trauma center case volume impacted procedural volume, we evaluated the annual patient-care interventions performed as a proportion of patients for whom they were potentially indicated. Specifically, we evaluated the proportion of patients who underwent:

1. A hemorrhage control procedure or blood product transfusion with a systolic blood pressure <100 mm Hg and heart rate >120 beats per minute.
2. Intensive care unit (ICU) admission with an ISS >15.
3. Intracranial pressure (ICP) monitor placement with a Head AIS of 3–5.
4. Operative intervention for an open femur or tibia fracture with an extremity AIS of 3–5.

We compared the proportions of procedures using bivariate statistics both across all quintiles and between Q1 and Q5. Need for trauma intervention was defined as a patient requiring at least 1 of the following interventions: transfusion of packed red blood cells or whole blood within 4 hours of arrival, discharge from ED to operating room ≤90 minutes from time of arrival, discharge from ED to interventional radiology ≤6 hours from arrival, admission to the ICU from ED with ICU length of stay ≥3 days, mechanical ventilation within 3 days of admission (excluding intubation events for operative anesthesia), and death within 60 hours of trauma center arrival.^15^

Analyses were performed using SAS 9.4 (SAS Institute, Inc., Cary, NC) and Stata 15.1 (StataCorp, College Station, TX). Institutional Review Board approval was obtained for this study, and it was deemed exempt as it relied on the secondary use and analysis of already collected data for quality improvement. The RECORD checklist was used to ensure proper reporting of methods, results, and discussion in accordance with ACS TQIP Appropriate Use Recommendations (Table S2, see https://links.lww.com/AOSO/A513).^16,17^

## RESULTS

**FIGURE 1.**
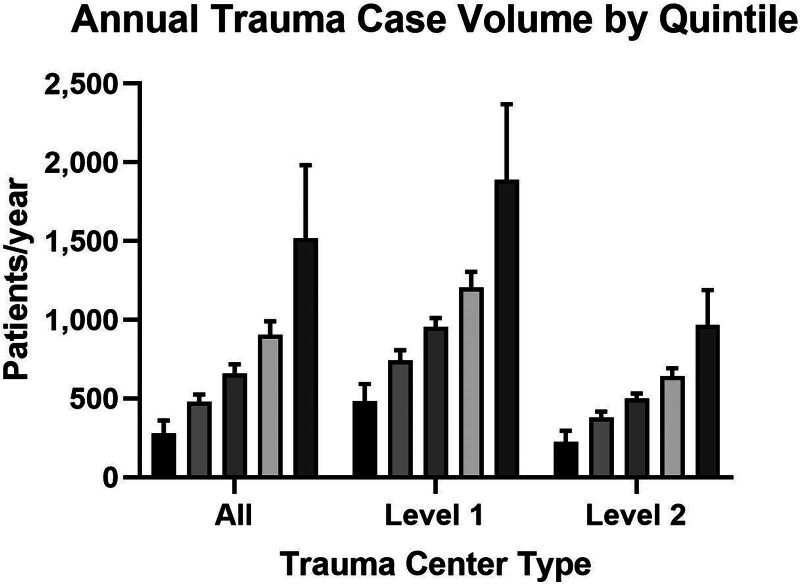
Graphs demonstrating annual trauma center ACS TQIP qualifying case volume by quintile for all trauma centers and stratified by levels 1 and 2.

We identified 1,902,005 patients eligible for study from 228 level 1 and 288 level 2 trauma centers. The trauma centers were divided into quintiles by mean case volume and stratified by verification level (Table 1). Overall, there was wide variation in case volume fulfilling National Trauma Data Standard inclusion criteria for ACS TQIP among both level 1 and 2 trauma centers (Fig. 1). Among level 1 trauma centers, the mean case volume of patients was nearly 4 times higher in the highest quintile relative to lowest. A similar difference was present in level 2 trauma centers. Only 40% of level 1 trauma centers treated >1200 patients per year meeting ACS TQIP inclusion criteria. When comparing between verification levels, the average level 2 trauma center case volume was higher than the average volumes at the lowest quintile level 1 institution. Case volume was associated with trauma center teaching status among level 1 trauma centers (*P* = 0.03), with higher volume centers having higher rates of university affiliation.

**TABLE 1.** Annual Trauma Center Case Volume

Type of Trauma Center	Quintile				
	1	2	3	4	5
Level 1, n hospitals	45	46	46	46	45
Volume, n patients					
Mean ± SD	484 ± 109	742 ± 66	953 ± 59	1205 ± 100	1888 ± 481
Median (IQR)	508 (429–565)	732 (694–799)	944 (908–999)	1207 (1109–1277)	1790 (1497–2084)
Minimum	195	626	854	1062	1392
Maximum	625	853	1062	1381	3562
Teaching status, %					
Community	37.8	43.5	19.6	21.7	13.3
Non-Teaching	2.2	8.7	4.4	6.5	0.0
University/Academic	57.8	45.7	76.1	69.6	84.4
Missing	2.2	2.2	0.0	2.2	2.2
Profit status*, %					
For-Profit	15.6	10.9	10.9	2.2	0.0
Nonprofit	84.4	89.1	87	97.8	100.0
Level 2, n hospitals	57	59	57	58	57
Volume, n patients					
Mean ± SD	224 ± 70	381 ± 37	499 ± 33	642 ± 51	966 ± 223
Median (IQR)	245 (178–282)	383 (348–415)	495 (473–524)	635 (601–685)	890 (798–1061)
Minimum	64	320	441	563	740
Maximum	316	440	562	739	1753
Teaching status, %					
Community	50.9	62.7	57.9	53.5	57.9
Non-Teaching	21.1	30.5	28.1	32.8	31.6
University/Academic	14.0	5.1	7.0	8.6	7.0
Missing	14.0	1.7	7.0	5.2	3.5
Profit status*, %					
For-Profit	19.3	13.6	22.8	20.7	24.6
Nonprofit	79.7	86.4	77.2	79.3	75.4

* Profit status of government was excluded to avoid identification due to small number.

Patient characteristics remained proportionally similar in most domains between trauma center case volume quintiles with some exceptions (Tables 2 and 3). Among level 1 trauma centers, mortality was highest in the first and fifth quintiles (8.4% and 8.0%). Mortality was lower among all level 2 trauma center quintiles (5.9%–6.9%) than level 1 quintiles (7.1%–8.4%). High-volume level 2 trauma centers had a higher mortality rate than low-volume level 2 trauma centers (6.8% vs 5.9%). Increasing case volume was associated with more patients ≤65 years old (67.0% vs 58.8%), presenting as inbound transfers (36.9% vs 22.5%), ISS ≥25 (18.7% vs 13.9%), or Glasgow coma scale motor score ≤2 (10.4% vs 8.1%) among level 1 trauma centers. Mechanism of injury also varied with case volume, as high-volume level 1 and 2 trauma centers had lower rates of falls (39.3% vs 52.2%) and higher rates of motor vehicle collision (35.5% vs 23.8%). Across all centers, penetrating trauma represented a low percentage of injuries, ranging by quintile from 5.2% to 6.6% of case volume at level 2 trauma centers and 11.3%–12.4% at level 1 trauma centers. Vital sign abnormalities, such as hypotension, tachycardia, and bradycardia, were present in 12.0% or fewer of patients across all quintiles.

**TABLE 2.** Patient Characteristics by Annual Case Volume and Percentage of Annual Case Volume for Level 1 Trauma Centers

Characteristic	Quintile										*P*
	1		2		3		4		5		
	Mean (SD)	%	Mean (SD)	%	Mean (SD)	%	Mean (SD)	%	Mean (SD)	%	
Patients	495 (131)	–	742 (111)	–	952 (154)	–	1205 (171)	–	1888 (520)	–	
Admitted	475 (129)	95.8	717 (108)	96.6	918 (149)	96.3	1161 (164)	96.4	1792 (485)	94.9	<0.001
Died	40 (18)	8.0	53 (19)	7.1	72 (26)	7.6	93 (27)	7.7	159 (60)	8.4	<0.001
Transfer out	8 (8)	1.7	13 (19)	1.8	14 (20)	1.5	13 (14)	1.1	17 (26)	0.9	<0.001
Transfer in	111 (99)	22.5	227 (120)	30.6	295 (133)	30.9	423 (174)	35.1	696 (321)	36.9	<0.001
Age category											
16–64 years	291 (105)	58.8	408 (99)	54.9	576 (158)	60.5	757 (173)	62.8	1264 (438)	67.0	<0.001
≥65 years	204 (103)	41.2	334 (106)	45.1	377 (147)	39.5	449 (150)	37.2	624 (198)	33.0	<0.001
Mechanism of injury											
Fall	259 (112)	52.2	393 (118)	53.0	451 (147)	47.3	540 (164)	44.8	742 (249)	39.3	<0.001
MVC	118 (58)	23.8	201 (68)	27.1	279 (88)	29.3	387 (105)	32.1	670 (257)	35.5	<0.001
Penetrating	56 (47)	11.3	56 (43)	7.6	97 (80)	10.2	118 (78)	9.8	234 (146)	12.4	<0.001
Hemodynamics											
SBP < 100 mm Hg	54 (28)	10.9	73 (29)	9.9	104 (40)	10.9	129 (50)	10.7	226 (108)	12.0	<0.001
HR > 120 bpm	32 (15)	6.4	45 (16)	6.0	64 (22)	6.8	86 (27)	7.1	142 (56)	7.5	<0.001
HR < 60 bpm	50 (23)	10.0	69 (25)	9.3	90 (30)	9.5	106 (38)	8.8	169 (82)	9.0	<0.001
Injury severity score											
ISS 9–15	323 (99)	65.2	465 (90)	62.7	583 (114)	61.2	715 (127)	59.3	1061 (252)	56.2	<0.001
ISS 16–24	104 (38)	20.9	164 (42)	22.0	218 (50)	22.9	289 (60)	24.0	475 (171)	25.2	<0.001
ISS ≥ 25	69 (29)	13.9	114 (33)	15.3	151 (44)	15.9	201 (57)	16.7	352 (150)	18.7	<0.001
AIS head/neck>2	171 (58)	34.5	253 (64)	34.0	314 (74)	33.0	410 (84)	34.0	643 (209)	34.1	<0.001
Glasgow coma scale											
GCS motor 1–2	40 (21)	8.1	61 (25)	8.2	85 (32)	9.0	115 (38)	9.5	196 (88)	10.4	<0.001
GCS motor 3–4	12 (7)	2.5	15 (6)	2.0	20 (9)	2.1	27 (11)	2.2	42 (20)	2.2	<0.001
GCS motor 5–6	416 (114)	83.9	628 (103)	84.7	809 (146)	85.0	997 (184)	82.7	1581 (432)	83.7	<0.001

AIS indicates abbreviated injury scale; bpm, beats per minute; GCS, Glasgow coma scale; HR, heart rate; ISS, injury severity score; mm Hg, millimeters of mercury; MVC, motor vehicle collision; SBP, systolic blood pressure.

**TABLE 3.** Patient Characteristics by Annual Case Volume and Percentage of Annual Case Volume for Level 2 Trauma Centers

Characteristic	Quintile										*P*
	1		2		3		4		5		
	Mean (SD)	%	Mean (SD)	%	Mean (SD)	%	Mean (SD)	%	Mean (SD)	%	
Patients	235 (77)	–	381 (81)	–	500 (79)	–	645 (99)	–	968 (257)	–	
Admitted	219 (74)	93.3	363 (79)	95.1	474 (76)	94.8	617 (99)	95.6	932 (254)	96.2	<0.001
Died	14 (7)	5.9	23 (11)	6.1	34 (14)	6.9	43 (15)	6.6	66 (23)	6.8	<0.001
Transfer out	16 (12)	7.0	18 (13)	4.6	18 (12)	3.7	26 (21)	4.1	22 (26)	2.3	<0.001
Transfer in	48 (52)	20.6	77 (75)	20.2	85 (66)	17.1	140 (86)	21.7	253 (145)	26.2	<0.001
Age category											
Age 16–64	116 (45)	49.4	188 (62)	49.3	270 (84)	54.1	349 (96)	54.0	510 (140)	52.7	<0.001
Age ≥65	119 (52)	50.6	193 (61)	50.7	229 (84)	45.9	297 (97)	46.0	458 (181)	47.3	<0.001
Mechanism of injury											
Fall	142 (60)	60.3	224 (70)	58.9	266 (96)	53.3	341 (111)	52.8	519 (193)	53.7	<0.001
MVC	53 (25)	22.7	91 (43)	24.0	136 (57)	27.2	186 (67)	28.8	277 (89)	28.6	<0.001
Penetrating	12 (11)	5.2	21 (19)	5.5	38 (36)	7.6	44 (36)	6.8	64 (39)	6.6	<0.001
Hemodynamics											
SBP <100 mm Hg	22 (11)	9.2	37 (16)	9.6	48 (20)	9.7	58 (20)	9.0	96 (44)	10.0	<0.001
HR > 120 bpm	12 (7)	5.0	20 (10)	5.2	29 (13)	5.9	39 (16)	6.0	56 (20)	5.8	<0.001
HR < 60 bpm	23 (10)	9.9	38 (15)	10.0	46 (15)	9.1	56 (17)	8.7	91 (41)	9.4	<0.001
Injury severity											
ISS 9–15	166 (57)	70.9	261 (65)	68.4	337 (70)	67.4	429 (86)	66.5	643 (198)	66.4	<0.001
ISS 16–24	42 (18)	18.0	73 (25)	19.0	100 (29)	20.0	131 (36)	20.3	197 (50)	20.3	<0.001
ISS ≥ 25	26 (13)	11.1	48 (22)	12.6	63 (24)	12.6	86 (29)	13.3	128 (41)	13.3	<0.001
AIS head/neck>2	67 (29)	28.6	111 (38)	29.1	145 (38)	28.9	199 (54)	30.8	298 (84)	30.7	<0.001
GCS score											
GCS motor 1–2	14 (9)	6.2	24 (15)	6.2	35 (18)	7.1	45 (18)	6.9	74 (30)	7.7	<0.001
GCS motor 3–4	5 (3)	2.0	8 (4)	2.0	10 (5)	2.1	15 (7)	2.3	18 (9)	1.9	<0.001
GCS motor 5–6	202 (73)	86.1	323 (77)	84.7	422 (96)	84.4	559 (100)	86.6	826 (247)	85.3	<0.001

AIS indicates abbreviated injury scale; bpm, beats per minute; GCS, Glasgow coma scale; HR, heart rate; ISS, injury severity score; mm Hg, millimeters of mercury; MVC, motor vehicle collision; SBP, systolic blood pressure.

**FIGURE 2.**
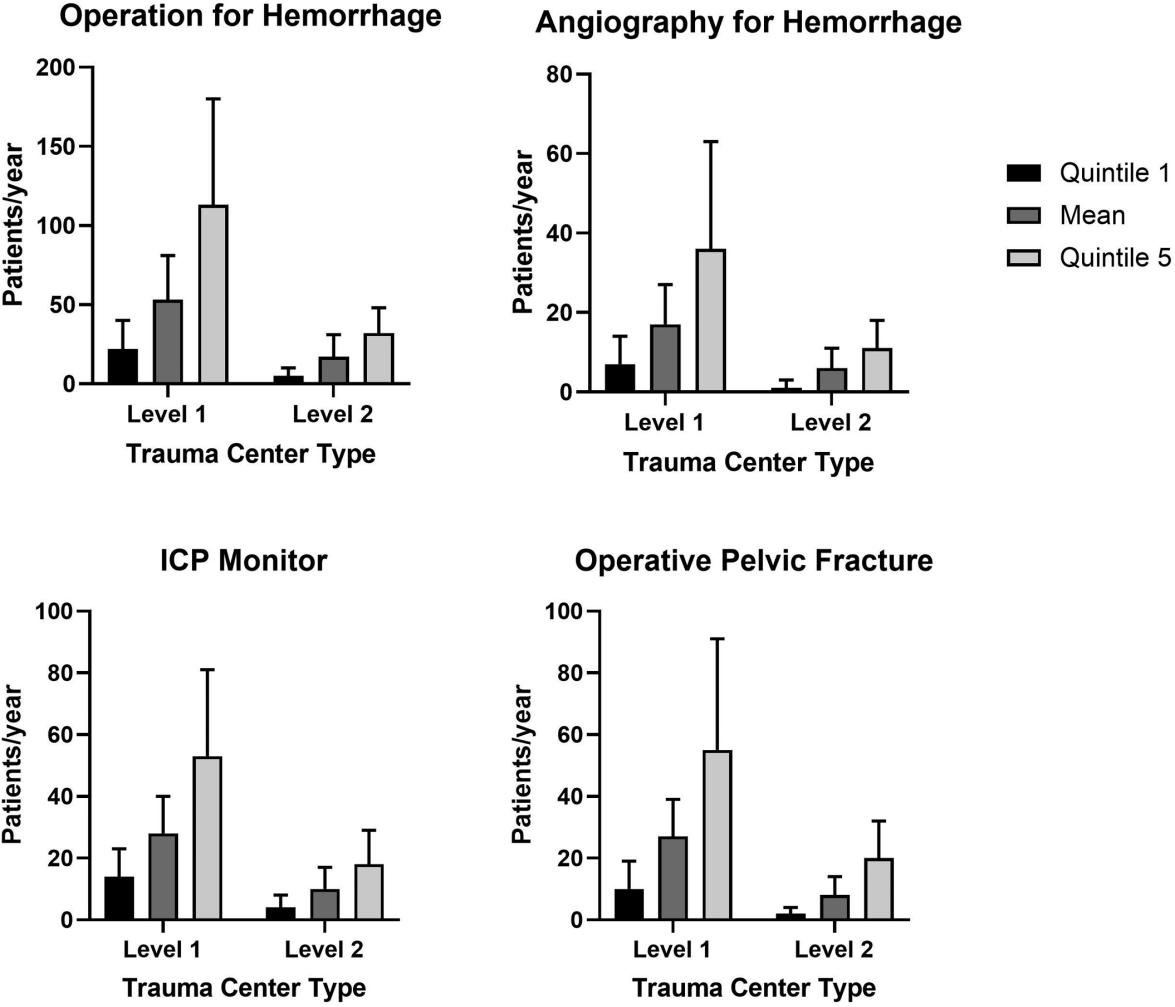
Graphs demonstrating annual trauma center procedural volume for level 1 and 2 trauma centers. Quintiles 1 and 5 are included with the mean. Procedures included are operation for hemorrhage, angiography for hemorrhage, ICP monitor placement, and operation for pelvic fracture.

There were significant differences in the absolute annual volume of patient-care interventions between high and low-volume trauma centers (Tables 4 and 5). Across all interventions evaluated, low-volume centers performed 50% fewer procedures than the overall trauma center average of the same verification level (Table S3, see https://links.lww.com/AOSO/A513). The highest volume trauma centers performed approximately 5 times as many procedures as the lowest quintile trauma centers for both the level 1 and level 2 cohorts (Fig. 2), For many procedures, the lowest quintile level 1 trauma centers performed <2 procedures monthly: operation for hemorrhage control (22 per year, 1.8 per month), angiography for hemorrhage control (7 per year, <1 per month), and pelvic fracture operations (10 per year, <1 per month) (Fig. 2). As a group, level 2 trauma centers had average overall rates of these procedures that were similar to the lowest quintile level 1 trauma centers: operation for hemorrhage (17 per year, 1.4 per month), angiography for hemorrhage (5 per year, <0.5 per month), and operative pelvic fracture operations (8 per year, <1 per month). The lowest volume quintile in level 2 trauma centers had <10 annual average patient-care interventions for many procedures considered essential for trauma center preparedness and verification.

**TABLE 4.** Patient-Care Interventions by Annual Volume and Percentage of Annual Case Volume for Level 1 Trauma Centers

Characteristic	Quintile										*P*
	1		2		3		4		5		
	Mean (SD)	%	Mean (SD)	%	Mean (SD)	%	Mean (SD)	%	Mean (SD)	%	
Patients	495 (131)	–	742 (111)	–	952 (154)	–	1205 (171)	–	1888 (520)	–	
Major trauma (NFTI)	165 (72)	33.3	230 (66)	31.0	343 (95)	36.0	439 (118)	36.5	672 (267)	35.6	<0.001
Operation for hemorrhage	22 (18)	4.4	28 (17)	3.7	44 (28)	4.6	58 (33)	4.8	113 (67)	6.0	<0.001
Angiography for hemorrhage	7 (7)	1.3	10 (6)	1.3	16 (10)	1.7	19 (11)	1.6	36 (27)	1.9	<0.001
Admit ICU	229 (90)	46.1	315 (93)	42.4	449 (120)	47.2	563 (154)	46.7	805 (287)	42.6	<0.001
Intubated	55 (33)	11.2	80 (36)	10.8	117 (70)	12.2	130 (62)	10.8	209 (109)	11.1	<0.001
Transfused PRBC	42 (28)	8.4	58 (28)	7.8	87 (42)	9.2	109 (50)	9.1	214 (117)	11.3	<0.001
ICP monitor	14 (9)	2.8	18 (11)	2.4	26 (12)	2.7	30 (16)	2.5	53 (28)	2.8	<0.001
Central line	26 (23)	5.3	38 (35)	5.2	51 (38)	5.3	55 (46)	4.6	119 (119)	6.3	<0.001
Chest tube	45 (24)	9.1	71 (25)	9.5	98 (37)	10.3	122 (41)	10.1	209 (88)	11.1	<0.001
BTAI	4 (3)	0.8	5 (4)	0.7	7 (5)	0.7	9 (6)	0.8	19 (11)	1.0	<0.001
Operative pelvic fracture	10 (9)	2.1	16 (10)	2.2	25 (12)	2.6	33 (16)	2.7	60 (36)	3.2	<0.001
Open fracture, femur	11 (8)	2.2	15 (8)	2.0	23 (13)	2.4	30 (15)	2.4	56 (33)	3.0	<0.001
Open fracture, tibia	21 (11)	4.2	30 (12)	4.1	43 (17)	4.5	57 (20)	4.7	100 (43)	5.3	<0.001

BTAI indicates blunt traumatic aortic injury; ICP, intracranial pressure; ICU, intensive care unit; NFTI, need for trauma intervention; PRBC, packed red blood cells.

**TABLE 5.** Patient-Care Interventions by Annual Volume and Percentage of Annual Case Volume for Level 2 Trauma Centers

Characteristic	Quintile										*P*
	1		2		3		4		5		
	N (SD)	%	N (SD)	%	N (SD)	%	N (SD)	%	N (SD)	%	
Patients	235 (77)	–	381 (81)	–	500 (79)	–	645 (99)	–	968 (257)	–	
Major trauma (NFTI)	58 (29)	24.6	101 (42)	26.5	147 (52)	29.5	198 (67)	30.7	305 (108)	31.5	<0.001
Operation for hemorrhage	5 (5)	2.1	10 (8)	2.6	17 (14)	3.3	21 (18)	3.3	32 (16)	3.4	<0.001
Angiography for hemorrhage	1 (2)	0.6	4 (4)	1.0	5 (5)	1.1	7 (7)	1.0	11 (7)	1.1	<0.001
Admit ICU	84 (44)	35.6	142 (52)	37.3	188 (62)	37.7	271 (77)	42.0	401 (150)	41.4	<0.001
Intubated	17 (13)	7.3	34 (19)	9.0	57 (40)	11.3	72 (44)	11.2	105 (48)	10.8	<0.001
Transfused PRBC	12 (9)	5.2	22 (15)	5.9	37 (24)	7.4	44 (30)	6.8	72 (33)	7.4	<0.001
ICP monitor	4 (4)	1.6	7 (6)	1.8	8 (7)	1.7	12 (9)	1.9	18 (11)	1.9	<0.001
Central line	7 (11)	2.9	14 (13)	3.6	22 (19)	4.5	27 (27)	4.2	42 (32)	4.3	<0.001
Chest tube	15 (9)	6.4	28 (15)	7.4	43 (19)	8.5	55 (27)	8.4	85 (33)	8.8	<0.001
BTAI	1 (1)	0.4	2 (2)	0.4	3 (2)	0.5	3 (2)	0.5	5 (3)	0.5	<0.001
Operative pelvic fracture	2 (2)	0.8	6 (5)	1.5	8 (6)	1.6	13 (10)	2.0	20 (12)	2.1	<0.001
Open fracture, femur	3 (3)	1.2	6 (4)	1.5	9 (6)	1.8	11 (7)	1.8	18 (9)	1.9	<0.001
Open fracture, tibia	8 (5)	3.2	13 (7)	3.4	20 (10)	4.0	26 (11)	4.0	41 (17)	4.2	<0.001

BTAI indicates blunt traumatic aortic injury; ICP, intracranial pressure; ICU, intensive care unit; NFTI, need for trauma intervention; PRBC, packed red blood cells.

The rate of patient-care interventions disproportionately increased relative to case volume when benchmarked against the number of patients for whom an intervention was potentially indicated (Table 6). For all interventions except ICU admission, the proportion of patients undergoing an intervention increased with case volume regardless of level 1 and level 2 trauma center status (*P* < 0.001). Of patients with an extremity AIS 3–5 presenting to a level 1 trauma center, the rate of undergoing operative repair of an open femur or tibia fracture was 39.9% higher at the highest quintile centers in relation to the lowest quintile (*P* < 0.001). Furthermore, patients with hypotension and tachycardia were more likely to undergo hemorrhage control procedures (31.8% vs 25.9%, *P* < 0.001) and transfusions (49.3% vs 40.0%, *P* < 0.001) at high volume level 1 trauma centers. High-volume level 1 trauma centers had lower rates of ICU admission among patients with an ISS >15 (64.1% vs 72.3%, *P* < 0.001). Level 2 trauma centers had results similar to level 1 trauma center across quintiles for the proportion of patients undergoing an intervention in the cohort for whom an intervention was potentially indicated. Collectively, high-volume trauma centers regardless of verification level performed absolutely and proportionality more procedural interventions for patients with potentially severe injury patterns.

**TABLE 6.** Annual Patient-Care Interventions Performed as a Proportion of Patients for Whom They Were Potentially Indicated

Characteristic	Quintile										*P*
	1		2		3		4		5		
	Mean (SD)	%	Mean (SD)	%	Mean (SD)	%	Mean (SD)	%	Mean (SD)	%	
A. Level 1 trauma center											
BP<100 and HR>120	39 (21)		53 (19)		78 (36)		96 (39)		178 (82)		
Operation or angiography for hemorrhage	10 (10)	25.9	13 (9)	25.1	23 (16)	29.0	28 (17)	28.6	57 (36)	31.8	<0.001
Transfused PRBC	16 (13)	40.0	22 (12)	41.3	36 (20)	45.7	43 (24)	44.8	88 (54)	49.3	<0.001
ISS>15	184 (67)		288 (73)		403 (84)		527 (112)		874 (350)		
Admit ICU	133 (53)	72.3	195 (54)	67.8	290 (66)	72.0	370 (82)	70.2	560 (217)	64.1	<0.001
Head AIS 3–5	151 (52)		224 (59)		273 (66)		354 (75)		549 (174)		
ICP monitor	14 (9)	9.0	17 (11)	7.8	25 (12)	9.2	29 (16)	8.3	52 (27)	9.4	<0.001
Extremity AIS 3–5	186 (67)		267 (82)		343 (88)		417 (92)		645 (195)		
Open fracture, femur or tibia	30 (17)	16.3	42 (17)	15.9	62 (26)	18.1	81 (31)	19.5	147 (70)	22.8	<0.001
B. Level 2 trauma center											
BP<100 and HR>120	15 (8)		24 (10)		36 (17)		44 (16)		69 (28)		
Operation or angiography for hemorrhage	2 (3)	16.1	5 (4)	20.9	8 (7)	22.5	10 (8)	22.1	17 (10)	24.6	<0.001
Transfused PRBC	5 (4)	31.8	9 (6)	36.3	15 (11)	40.5	17 (11)	38.3	30 (16)	43.4	<0.001
ISS >15	71 (30)		126 (46)		175 (53)		228 (63)		343 (90)		
Admit ICU	45 (22)	62.7	83 (33)	66.4	114 (40)	64.9	158 (47)	69.1	243 (78)	70.7	<0.001
Head AIS 3–5	60 (26)		100 (35)		129 (34)		178 (49)		266 (78)		
ICP monitor	4 (4)	6.0	7 (6)	6.6	8 (6)	6.4	12 (8)	6.6	18 (11)	6.6	<0.001
Extremity AIS 3–5	101 (43)		156 (54)		156 (54)		255 (82)		391 (150)		
Open fracture, femur or tibia	10 (6)	10.1	18 (9)	11.4	28 (14)	13.6	36 (16)	13.9	56 (24)	14.3	<0.001

BTAI indicates blunt traumatic aortic injury; ICP, intracranial pressure; ICU, intensive care unit; PRBC, packed red blood cells.

## DISCUSSION

This evaluation of national trauma center case volume variation has 3 principal findings. First, the identification of wide variation in case volume may suggest a potential mismatch between trauma center distribution and population needs. Second, low absolute case volume and procedural volume at some trauma centers may have implications for the optimization of patient outcomes, resource distribution, and clinical skills maintenance. Third, high-volume trauma centers perform more patient-care interventions as a proportion of patients for whom they are potentially indicated. Collectively, these results demonstrate an opportunity for system improvement through a more nuanced approach to trauma center resource allocation and oversight.

We found particularly low patient volumes at many level 1 and 2 trauma centers which has 2 potential explanations: low population density as seen in rural areas or areas with oversaturation of trauma centers for a given population density. In rural trauma centers, the low volume may still indicate the appropriate distribution of trauma centers as extensive prior work has demonstrated improved outcomes by minimizing the distance from injury occurrence to the nearest level 1 or 2 trauma center.^18–20^ Conversely, lower case volumes in more densely populated areas may be driven by over-proliferation of trauma centers. There is evidence that novel economic drivers are contributing to an oversupply of level 1 and 2 trauma centers in adequately saturated communities.^7^ When trauma center proliferation is guided by incentives beyond broadening access, costs rise, established trauma centers may suffer financially, and patient outcomes may be affected as the volume-outcome relationship is harmed.^21^ Optimal trauma center distribution should result in a relatively consistent patient case volume with mild-to-moderate variation for differences in regional population density. However, our findings demonstrating a fourfold difference in case volume from the highest to lowest quintiles suggest opportunities for more efficient trauma resource allocation.

Low absolute rates of case volume and procedural volume in low-volume trauma centers may have implications for patient outcomes and the threshold at which to perform procedures. Prior work evaluating the relationship between trauma center case volume and outcomes has yielded conflicting results. However, the overall body of evidence suggests that higher trauma case volume is associated with improved outcomes at the facility and provider level.^12,22–24^ This benefit has been demonstrated at the level of individual procedures, such as trauma laparotomy.^25^ Furthermore, prior multidimensional approaches for evaluating trauma center quality and patient safety, including metrics such as time to the operating room for gunshot wounds, have demonstrated that the highest-performing centers treat higher volumes of severely injured patients.^26^ In this context, wide variation in case volume among level 1 and 2 trauma centers may have implications surrounding the quality of care provided. Specifically, low-volume trauma centers may experience unique challenges in achieving quality or metric benchmarks. This must be balanced with the role of rural trauma centers representing an important geographic safety net of timely, life-saving trauma care.^27^

Case volume differences between trauma center verification levels may have implications for resource availability and utilization. With the exception of the expectation of a research commitment and an annual threshold of patient volume, almost all of the resources expected to be available at a level 1 trauma center are also mandated at a level 2 trauma center.^9^ However, prior work has demonstrated improved outcomes at level 1 over level 2 trauma centers for complex injuries, such as high-grade liver injury or unstable pelvic ring fracture.^28–30^ Potential explanations for this association include higher resource availability and increased familiarity of providing care for these complex cases at high-volume trauma centers. Our results extend this work by describing the inconsistent relationship between case volume and verification level. We found that the lowest quintile level 1 trauma centers have lower mean case volume than the average level 2 trauma centers. Expectations of improved outcomes for high-complexity injury may be unrealized when level 2 trauma centers already have more experience with complex cases. Regardless of resource availability, hemorrhage control operations performed at low volume level 1 centers (22 annually, 1 every 16 days) may be challenging with regard to efficient time to the operating room and conduct of the operation when compared to the highest volume level 2 centers (32 annually, 1 every 11 days) that have more opportunities for repetition and familiarity with their response.

High-volume trauma centers performing more patient-care interventions relative to the number of potentially eligible patients may imply important differences in patterns of care between low and high-volume trauma centers. This difference is troubling as the goal of the ACS COT verification system is to assure the provision of timely, appropriate, high-quality trauma care to all patients regardless of which level 1 or 2 trauma center they present to. It is possible that trauma centers performing procedures most frequently are more comfortable performing the intervention in circumstances where the decision to perform the intervention or not has relative equipoise. However, the significant differences in rates of hemorrhage control procedures and blood product transfusion among patients with hypotension and tachycardia are hard to interpret without wondering if life-saving care is being delayed. The converse finding that ICU utilization is reduced in higher-volume trauma centers could have a few explanations. It is possible that high-volume trauma centers are more familiar with a certain type of problem and feel comfortable managing the patient in a general care unit. High-volume trauma centers may also have lower ICU bed availability due to overcrowding leading to potential undertriage of patients to the ICU who have an indication for this resource. It is unlikely that differences in patient characteristics and complexity are responsible for our finding of patient-care interventions increasing with case volume. Patient characteristics and complexity remained generally similar in most contexts independent of case volume – especially among the lower 80% of trauma centers. It must be acknowledged, however, that prior work has demonstrated that some trauma centers with very high procedural volumes are low in total case volume.^31^ Hence, procedural volume, case volume, and intervention rates are all important to understanding the performance of any individual trauma center.

Low procedural volume has implications for surgical trainee education and ongoing maintenance of clinical skills. The Accreditation Council for Graduate Medical Education mandates general surgery residents perform 10 operative and 40 nonoperative trauma cases during residency.^32^ In the context of prior research demonstrating inadequate resident exposure to operative trauma, the disparity in procedural volume between trauma centers in this study highlights a surgical training problem.^33–35^ The mean duration of time general surgery residents rotate on trauma surgery is 6.0 months.^36^ For residents affiliated with low-volume level 1 trauma centers performing <2 hemorrhage control operations per month, it might be challenging to achieve the graduation minimum. These findings have widespread implications for workforce preparedness, as the majority of low-volume trauma centers were teaching facilities. Furthermore, while the Accreditation Council for Graduate Medical Education threshold represents the minimum allowable case volume to achieve licensure, it may not represent the case volume needed to achieve operative and clinical self-efficacy when transitioning into independent practice. This may be contributing to the increasing number of trainees pursuing operative fellowship training.^37^ Beyond resident education, these results also have implications for the maintenance of clinical skills for surgeons in independent practice. Without adequate clinical exposure, surgeons at low-volume trauma centers may be at risk of lapses in clinical competency.^38,39^ This may represent an opportunity for enhanced availability of simulation technology for trainee and general surgery continuing education.

These findings should be interpreted within the context of the study’s limitations. First, the study only captures trauma patients who meet ACS TQIP inclusion criteria, notably excluding lower AIS acuity trauma patients. As these results describe case volume variation of only moderate and high acuity patients, this evaluation should not be used to understand variation in global operations between trauma centers. However, this population does represent patients with elevated care needs for which the enhanced resources and readiness of level 1 and 2 trauma centers are designed to support. Thus, this population is appropriate for considering the implications of case volume variation in relation to the function of high verification level trauma centers. The external validity of this study is supported by work describing similar patient characteristics and acuity across trauma centers using ACS TQIP data.^40^ Second, this study is limited by the lack of long-term outcomes data. While prior literature suggests that case volume variation may imply variation in outcomes, this potential implication would be further strengthened if more granular long-term outcomes data beyond unadjusted mortality and complications was available. Third, the contextualization of this study is limited by the absence of geographic data describing the rurality or urbanicity of trauma centers. To fully appreciate the implications of case volume variation, it would be appropriate to account for variance in population density. A low-volume level 1 trauma center that alone services a vast expanse of rural territory may represent a better allocation of trauma resources than a similar volume level 2 trauma center in an urban setting with multiple surrounding level 1 trauma centers. These findings motivate further investigations incorporating case volume with population density to understand the appropriateness of trauma resource distribution.

## CONCLUSIONS

This study has several important implications for trauma systems. First, wide variation in case volume between trauma centers may suggest a mismatch between their distribution and population needs. Second, low patient and procedural volumes at some trauma centers may have implications for patient outcomes, surgical training, and maintenance of clinical skills. Finally, disproportionately higher rates of procedural interventions among high-volume trauma centers suggest important differences in clinical practice patterns between high and low-volume centers. Collectively, these results may inform the ongoing development and regulation of trauma systems. As trauma systems continue to proliferate and evolve, optimal resource allocation is critical for improving patient access and quality of care.

## ACKNOWLEDGMENTS

This study adhered to ACS Trauma Quality Programs Appropriate Use Recommendations. The ACS TQIP participant use file remains the full and exclusive copyrighted property of the American College of Surgeons. The American College of Surgeons is not responsible for any claims arising from works based on the original Data, Text, Tables, or Figures.

## Supplementary Material
